# Effect of the Niobium-Doped Titanium Oxide Thickness and Thermal Oxide Layer for Silicon Quantum Dot Solar Cells as a Dopant-Blocking Layer

**DOI:** 10.1186/s11671-020-3272-8

**Published:** 2020-02-10

**Authors:** Ryushiro Akaishi, Kohei Kitazawa, Kazuhiro Gotoh, Shinya Kato, Noritaka Usami, Yasuyoshi Kurokawa

**Affiliations:** 1 Material Process Engineering, Graduate School of Engineering, Nagoya University, Furo-cho, Chikusa-ku, Nagoya, 464-8603 Japan; 2 Department of Electrical and Mechanical Engineering, Nagoya Institute of Technology, Syouwa-ku, Nagoya, 466-8555 Japan

**Keywords:** Silicon quantum dot, Solar cell, Nb-doped titanium oxide, Amorphous silicon oxide, Thermal oxide

## Abstract

Silicon quantum dot (Si-QD) embedded in amorphous silicon oxide is used for p-i-n solar cell on quartz substrate as a photogeneration layer. To suppress diffusion of phosphorus from an n-type layer to a Si-QD photogeneration layer, niobium-doped titanium oxide (TiO_x_:Nb) is adopted. Hydrofluoric acid treatment is carried out for a part of the samples to remove the thermal oxide layer in the interface of TiO_*x*_:Nb/n-type layer. The thermal oxide acts as a photo-generated carrier-blocking layer. Solar cell properties using 10-nm-thick TiO_*x*_:Nb without the thermal oxide are better than those with the thermal oxide, notably short circuit current density is improved up to 1.89 mA/cm^2^. The photo-generated carrier occurs in Si-QD with quantum confinement effect. The 10-nm-thick TiO_*x*_:Nb with the thermal oxide layer effectively blocks P; however, P-diffusion is not completely suppressed by the 10-nm-thick TiO_*x*_:Nb without the thermal oxide. These results indicate that the total thickness of TiO_*x*_:Nb and thermal oxide layer influence the P-blocking effect. To achieve the further improvement of Si-QD solar cell, over 10-nm-thick TiO_*x*_:Nb is needed.

## Introduction

Silicon quantum dot (Si-QD) has been studied to realize over 40% efficiency solar cells [1–4]. The single-junction Si solar cell exceeding 26% was recently produced [5], which is quite reaching the theoretical limit, about 30% [6]. The other approaches are essential for further improvement of the conversion efficiency. Tandem configuration is one of the solutions to overcome the limit by using the multi-junction with several bandgaps [7–9]. Si-QD is one of the candidates for the top cell on the tandem solar cell since the bandgap depending on its size can be tuned due to the quantum confinement effect [10–14]. Besides, Si-QD has some advantages originated from the element characteristics: earth-abundant, non-toxic, and easy-application of industries. In this study, Si-QD multilayer structure (Si-QDML) was used to fabricate the Si-QDs, which is embedding Si-QDs in wide-gap materials [15–17].

The p-i-n solar cell structure using Si-QDML with silicon dioxide (SiO_2_) has been fabricated and been measured current density-voltage (*J*-*V*) characteristics [18, 19]. The SiO_2_ matrix can reduce dangling bonds of the Si-QD surface, leading to a high level of surface passivation of Si-QD [20]. One of the solar cell structures had a high open-circuit voltage (*V*_OC_) of 492 mV. However, short-circuit current density (*J*_SC_) was very poor due to the low tunneling probability of photo-generated carriers, which is caused by the large band offset between crystalline Si and SiO_2_ [1, 8]. Also, a quite large series resistance originated from the high sheet resistance of n-type Si-QDML was observed. To solve these problems, we proposed to use the Si-QDML with oxygen-deficient amorphous silicon oxide to increase the tunneling probability of photo-generated carriers [21], leading to an increase in *J*_SC_. Additionally, highly doped n-type polycrystalline silicon (n^++^-poly-Si) was adopted as a conductive layer to decrease the resistance, bringing the good enhancement of *J*_SC_ and fill factor (FF). Meanwhile, diffusing the P from n-type layer into the Si-QDML causes the deterioration of the film quality. Thus, the P-blocking layer without falling to the electrical and optical properties is necessary.

Niobium-doped titanium oxide (TiO_*x*_:Nb) is one of the promising materials for a P-blocking layer. TiO_*x*_:Nb is one of the electron selective contacts for crystalline silicon and can keep low resistivity even after annealing at high temperatures [22]. We have investigated the Si-QDs for the solar cell application [11, 16, 23–27], and a high *V*_OC_ of 529 mV was finally obtained using the 2-nm-thick TiO_*x*_:Nb [28]. Although, suppression of P diffusion is crucial to realize the higher performance of the Si-QD solar cells, the effect of P diffusion on the Si-QD solar cells is not fully understood.

In this paper, the effect of TiO_*x*_:Nb thickness, influencing on the P-diffusion, and the solar cell properties using Si-QDML with silicon oxide matrix were investigated. Moreover, the thermal oxide layer was formed on the n^++^-poly-Si during the fabrication process, affecting the P-diffusion and solar cell properties. The effects of the thermal oxide layer were also discussed here.

## Experimental Methods

To analyze the P-depth profile, Si-QDML/TiO_*x*_:Nb/n^++^-poly-Si structure was fabricated on quartz substrates. Prior to depositing heavily P-doped hydrogenated amorphous silicon (n^++^-a-Si:H) layer, the quartz substrates were cleaned in an ultrasonic bath containing an organic solvent. n^++^-a-Si:H thin film was prepared by plasma-enhanced chemical vapor deposition (PECVD) with a frequency of 27.12 MHz (ULVAC Inc., CME-200 J). The layer thickness of the n^++^-a-Si:H was about 500 nm. The deposition temperature, chamber pressure, and the radio frequency (RF) power were 195 °C, 25 Pa, and 32.5 mW/cm^2^, respectively. The films were annealed at 900 °C for 30 min under forming gas atmosphere to form n^++^-poly-Si by a lamp furnace (ADVANCE RIKO Inc., MILA-5050). During the annealing process, the thermal oxide layer was spontaneously formed on the n^++^-poly-Si. One of the samples was dipped in the 5 % HF solution for 1 min to remove the ultra-thin thermal oxide layer. 2 or 10-nm-thick TiO_*x*_:Nb was immediately deposited by RF magnetron sputtering after HF treatment. The deposition temperature, argon gas flow rate and pressure, and RF power were room temperature, 50 sccm, 0.2 Pa, and 137 mW/cm^2^, respectively. Subsequently, a-SiO_*x*_:H and a-SiO_*y*_:H were alternately deposited by the PECVD for a Si-rich layer and a barrier layer, respectively. The SiH_4_/CO_2_ ratio of the Si-rich layer and O-rich layer were 1.0 and 0.16, respectively; therefore, *y* was larger than *x*. The stacking cycle was 30 periods. The deposition temperature, chamber pressure, and RF power were the same as the n^++^-a-Si:H deposition condition. The samples were annealed at 900 °C for 30 min under forming gas atmosphere to form Si-QDs in Si-rich layers.

**Fig. 1:**
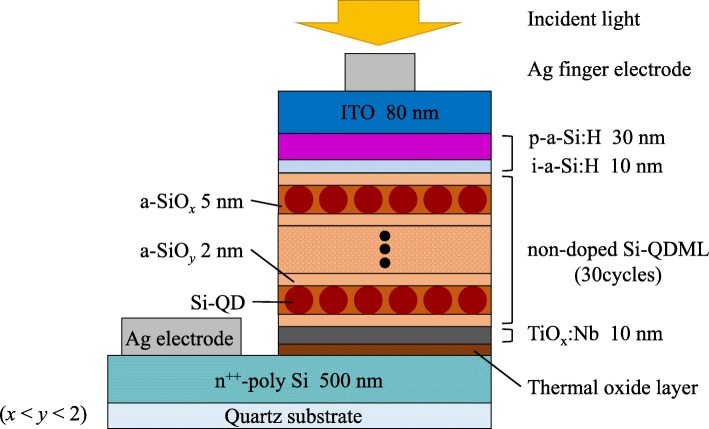
Schematic cross-sectional diagram of Si-QD solar cell structure, not in scale. A part of the samples was removed the thermal oxide layer

We also fabricated p-i-n solar cells on quartz substrates. Figure 1 shows the schematic diagram of the solar cell structure. The fabrication process from substrate cleaning to a-SiO_*x*_:H/a-SiO_*y*_:H bilayers annealing was the same as the samples for P-depth analysis. The thicknesses of TiO_x_:Nb, a-SiO_*x*_:H, and a-SiO_*y*_:H were kept at 10, 5, and 2 nm, respectively. The hydrogen atoms were injected into the samples so as to reduce the dangling bonds in Si-QDML by hydrogen plasma treatment with a frequency of 60 MHz (KATAGIRI ENGINEERING CO.). The process temperature, pressure, and time were 225 °C, 600 Pa, and 60 min, respectively. 10-nm-thick non-doped hydrogenated amorphous silicon (i-a-Si:H) and 30-nm-thick boron-doped hydrogenated amorphous silicon (p-a-Si:H) bilayer was deposited by the PECVD. An indium tin oxide (ITO) layer was deposited by RF sputtering, and finally, Ag electrode was evaporated.

The Si-QDML/TiO_*x*_:Nb/n^++^-poly-Si was directly observed by high-resolution transmission electron microscopy (HRTEM) using a JEOL JEM-ARM200F. The accelerating voltage was set at 200 kV. The depth profile of P was analyzed by time-of-flight secondary ion mass spectroscopy (TOF-SIMS) and secondary ion mass spectroscopy (SIMS). Sputtering was accomplished by Bi^3+^ at 30 kV in TOF-SIMS and done by Cs^+^ at 5 kV in SIMS. *J*-*V* measurement was carried out under the solar simulator illumination at AM1.5G, 100 mW/cm^2^, and room temperature. External quantum efficiency (EQE) was also carried out under the constant photon irradiation at room temperature. From the EQE and the reflectance of the solar cell, the internal quantum efficiency (IQE) was calculated using the following equation.

1 $$ IQE\left(\lambda \right)=\frac{EQE\left(\lambda \right)}{1-R\left(\lambda \right)} $$

The layer thickness was characterized by a spectroscopic ellipsometer (J. A. Woollam Co., M-2000DI-Nug).

## Results and Discussion

**Fig. 2:**
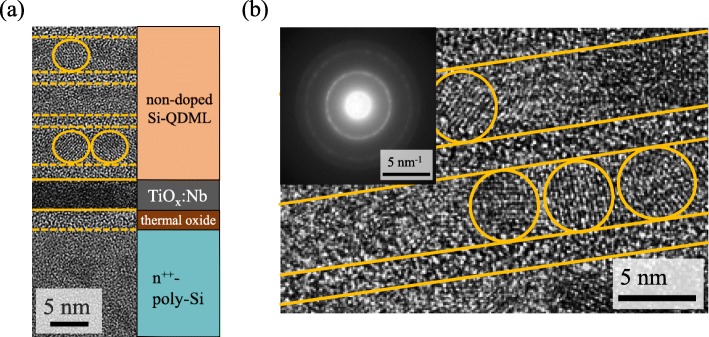
Cross-sectional HRTEM images of **a** Si-QDML/TiO_*x*_:Nb/thermal oxide/n^++^-poly-Si structure and **b** Si-QDML. The inset in (b) is the electron diffraction pattern

Figure 2 a shows the HRTEM image of Si-QDML/TiO_*x*_:Nb/n^++^-poly-Si structure. Note that for this sample HF treatment was not carried out before TiO_*x*_:Nb deposition. A brighter layer can be seen between TiO_*x*_:Nb and n^++^-poly-Si, indicating the thermal oxide layer was formed during the n^++^-a-Si:H process. Figure 2 b shows the magnified cross-sectional HRTEM image of Si-QDML. The inset in Fig. 2b shows the electron diffraction pattern of Si-QDML. It was confirmed that the multilayer structure was successfully fabricated. The fringes, originated from the Si-QDs crystalline phase, were only formed in the Si-rich layer. From the diffraction pattern, the lattice constant was calculated at 5.40 Å, which is in good agreement with the crystalline Si lattice constant of 5.43 Å. The sizes of Si-QDs were almost equal to the Si-rich layer thickness (~ 5 nm), suggesting that the size controlling was successfully achieved.

**Fig. 3:**
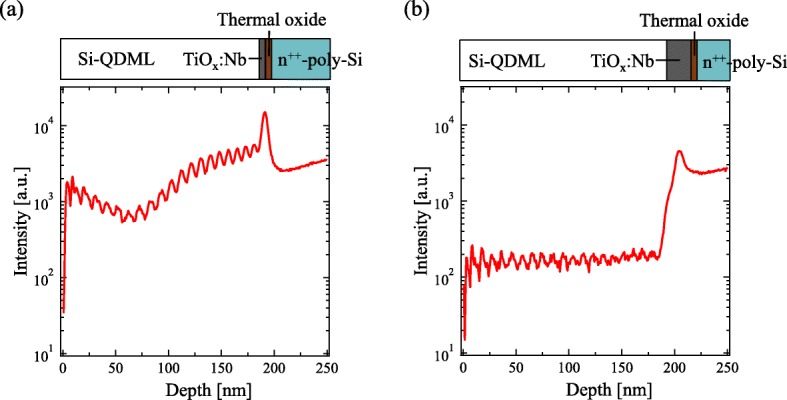
Depth profile of phosphorus atoms in Si-QDML/TiO_*x*_:Nb/thermal oxide/n^++^-poly-Si structure using **a** 2-nm-thick TiO_*x*_:Nb and **b** 10-nm-thick TiO_*x*_:Nb

**Fig 4:**
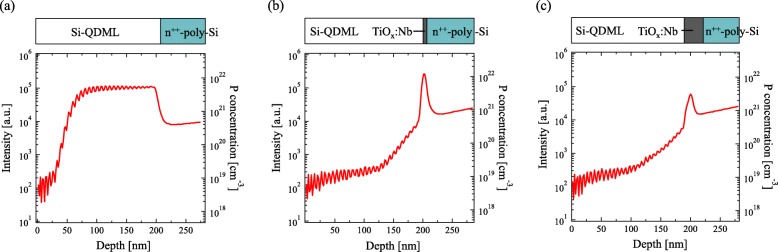
Depth profile of phosphorus atoms in **a** Si-QDML/n^++^-poly-Si and Si-QDML/TiO_*x*_:Nb/n^++^-poly-Si structure using **b** 2-nm-thick TiO_*x*_:Nb and **c** 10-nm-thick TiO_*x*_:Nb

Figure 3 shows the P depth profile of the Si-QDML/TiO_*x*_:Nb/thermal oxide/n^++^-poly-Si structure employing (a) 2-nm-thick and (b) 10-nm-thick TiO_*x*_:Nb. The Si-QDML was 20 cycles of 10-nm-thick Si-rich layer and 1-nm-thick barrier layer. The wave-like periodic intensities in the Si-QDML region are caused by the matrix effect and represent the multilayer structure. Since the detection sensitivity is changed due to the different ionization rate depending on the buried matrix, undulations of the intensity are observed for the multilayer structures [29]. The intensity of P ions between Si-QDML and n^++^-poly-Si was not decreased in 2-nm-thick TiO_*x*_:Nb sample, indicating the P diffusion occurred. On the contrary, for the sample employing the 10-nm-thick TiO_*x*_:Nb, the intensity of P ions in the Si-QDML was suppressed by an order of magnitude compared to that in n^++^-poly-Si. The results suggest that the thicker TiO_*x*_:Nb is effective for blocking the interdiffusion of P. Figure 4 shows the depth profile of P intensity and P concentration on (a) the Si-QDML/n^++^-poly-Si and Si-QDML/TiO_*x*_:Nb/n^++^-poly-Si structure employing (b) 2-nm-thick and (c) 10-nm-thick TiO_*x*_:Nb. In this figure, the Si-QDML was 30 cycles of 5-nm-thick Si-rich layer and 2-nm-thick barrier layer. We emphasize that HF treatment was performed in these samples before the TiO_*x*_:Nb deposition, therefore the thermal oxide was removed. In (Fig. 4a), no reduction of P intensity in Si-QDML region was observed. The P intensity in Si-QDML was higher than that in n^++^-poly Si in (Fig. 4a). A similar tendency was observed in (Fig. 3a). It is possible that the defects in Si-QDML worked as gettering sites for P [30]. In contrast, the intensity of P in Si-QDML with 2 and 10-nm-thick TiO_*x*_:Nb layer was 2 orders of magnitudes less than that in n^++^-poly-Si, as you see in Fig. 4 b and c. The 10-nm-thick TiO_*x*_:Nb without the thermal oxide layer did not completely block the P interdiffusion. In (Fig. 4c), the concentration of diffused P atoms was less than 3 × 10^20^ cm^−3^ and the diffusion length was around 100 nm. However, without the TiO_*x*_:Nb and thermal oxide interlayer (Fig. 4a), the concentration of diffused P atoms was around 5 × 10^21^ cm^−3^ and the diffusion length was more than 150 nm, suggesting that the 10-nm-thick TiO_*x*_:Nb influences the P-blocking effect, although it was not sufficient. The P intensity profile of 10-nm-thick TiO_*x*_:Nb sample was almost identical to that of the samples with 2-nm-thick TiO_x_:Nb, indicating that the thermal oxide layer on n^++^-poly-Si also contributes to the P-blocking [31]. Since the P-blocking can be realized by inserting TiO_*x*_:Nb layer between Si-QDML and n^++^-poly-Si, we tried to apply the 10-nm-thick TiO_*x*_:Nb to our solar cell structure.

**Fig. 5:**
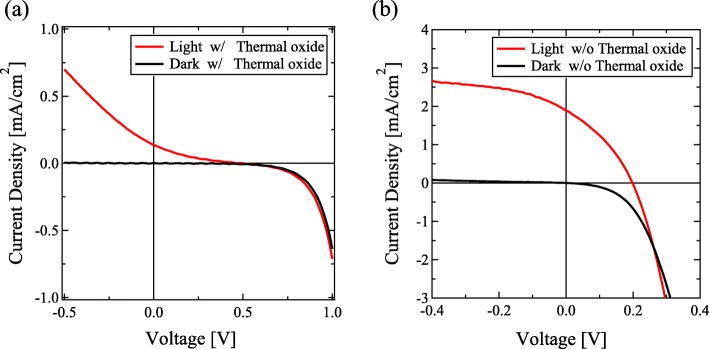
*J*-*V* characteristics of the solar cell structure **a** with thermal oxide and **b** without thermal oxide. 10-nm-thick TiO_*x*_:Nb was deposited in this solar cell

**Fig. 6:**
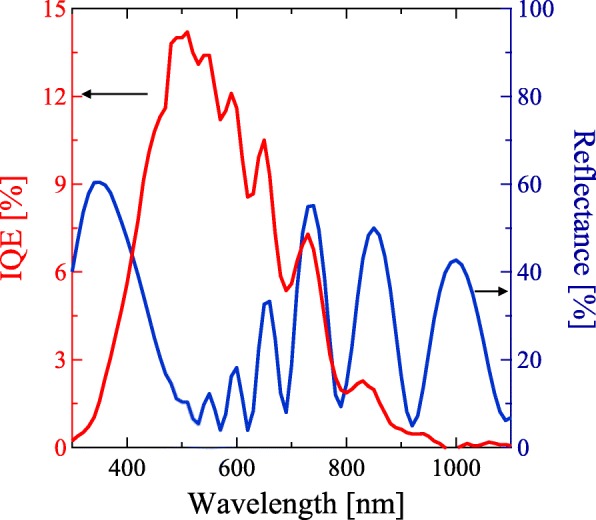
Internal quantum efficiency and reflectance versus wavelength for the fabricated solar cell without thermal oxide layer. The IQE and reflectance were drawn with red and blue, respectively. The TiO_*x*_:Nb layer thickness was 10 nm

Figure 5 shows the *J*-*V* characteristics of the Si-QDML solar cells (a) with and (b) without the thermal oxide interlayer. The *J*_SC_, *V*_OC_, FF, and conversion efficiency are summarized in Table 1. We did not use the interdiffusion process in our solar cell. Hence the effects of the defects formed by the dopant interdiffusion, which is one of the problems for the former Si-QD solar cell structure, can be neglected. In (Fig. 5a), the S-shaped curve was observed in the forward bias condition in the sample with the thermal oxide. In contrast, the *J*-*V* curve of the solar cell without the thermal oxide showed rectifying properties (see in Fig. 5b). Considering the results, we suggest that the photo-generated carriers were blocked by the thermal oxide layer, whereas photo-generated carriers were efficiently collected by removing the thermal oxide layer, which results in the S-shaped diode curve. The *J*_SC_ was drastically increased from 0.137 to 1.89 mA/cm^2^. Furthermore, the series resistance under the illumination was significantly decreased from 11 kΩ∙cm^2^ to 59 Ω∙cm^2^ after HF treatment. On the other hand, the decrease in *V*_OC_ was observed for the solar cell with the HF treatment possibly due to the enhanced P-diffusion as shown in Figs. 3 and 4. In the case of a-Si thin film solar cells, pn junction does not have enough photovoltaic effect since doped a-Si layers have high defect density and photo-generated carriers were recombined at the pn interface immediately. Hence, to avoid such a leakage current due to recombination at the pn interface, an undoped a-Si layer has been inserted. Our Si-QDML solar cell also has p-i-n structure. Unintentionally, in the case of without thermal oxide layer, undoped Si-QDML was changed into P-doped Si-QDML. P-doped Si-QDML should have larger defect density compared with undoped Si-QDML since Si-QDML includes an amorphous phase. Leakage current at the p-a-Si:H/P-doped Si-QDML interface due to carrier recombination degraded *V*_OC_. The 10-nm-thick TiO_*x*_:Nb with thermal oxide layer successfully suppressed the P diffusion, leading to a high *V*_OC_ of 502 mV. On the other hand, only 10-nm-thick TiO_*x*_:Nb did not completely block the P diffusion, as you see in (Fig. 4c). Therefore, *V*_OC_ degradation occurred. For further improvement of solar cell properties, we suggest that depositing thicker TiO_*x*_:Nb is necessary to prevent P atoms from diffusion into the Si-QDML. As mentioned above, the total thickness of TiO_*x*_:Nb and thermal oxide layer influences the P diffusion. From these results, thicker TiO_*x*_:Nb than 10 nm may enhance solar cell property. Figure 6 shows the IQE of the Si-QD solar cell without the thermal oxide layer. The reflectance spectrum of the solar cell was also shown. The periodic intensity change seen in the IQE is suggested to be the influence of interference by the solar cell structure due to using the flat substrate. We considered that the interference occurred in the thin-film solar cell region, mainly the reflection from n^++^-poly-Si/quartz substrate. The refractive index on Si, approximately 3.4, is quite different from that on quartz, 1.5 [32, 33]. The reflection waves interacted with the incident light, hence the wave periodic reflectance was observed. A similar trend of reflectance spectrum with several hundred-nanometer-thick silicon thin films has been reported [34, 35]. We suggest that the texturized substrate will disappear such an interaction. Our former research showed the IQE spectrum without any interference using the rough surface substrate [28]. The edge of the IQE spectrum was located about 1000 nm (being equal to 1.24 eV), corresponding to the PL peak (see our former report in ref. [21]). The IQE edge did not match with the absorption edge of general bulk-silicon and amorphous silicon, indicating that the carrier generation occurred in silicon nanocrystals with quantum confinement effect.

**Table 1:** Characteristics of silicon quantum dot solar cells with or without the thermal oxide interlayer

	*J*_SC_ [mA/cm^2^]	*V*_OC_ [V]	FF	Efficiency [%]
With thermal oxide interlayer	0.137	0.502	0.148	0.010
Without thermal oxide interlayer	1.89	0.198	0.340	0.127

## Conclusion

We adopted the TiO_*x*_:Nb layer as a P-blocking layer on a Si-QD solar cell. The dependence of TiO_*x*_:Nb thickness and the existence of the thermal oxide layer on the n-type layer were investigated and the solar cell properties were characterized. The diffusion of P atoms into Si-QDML was suppressed by the 10-nm-thick TiO_*x*_:Nb and ultrathin thermal oxide interlayer. The concentration of diffused P atoms in 10-nm-thick TiO_*x*_:Nb without the thermal oxide layer was about 3 × 10^20^ cm^−3^, which was over one magnitude less than that without TiO_*x*_:Nb and thermal oxide layer. Besides, the diffusion length decreased from 150 to 100 nm. These declines suggest that the 10-nm-thick TiO_*x*_:Nb influences the P-blocking effect, although the P diffusion was not completely blocked. The solar cell properties with 10-nm-thick TiO_*x*_:Nb were measured. The *J*-*V* curve of the solar cell with the thermal oxide was S-shape, whereas that without thermal oxide was improved, especially *J*_SC_ (from 0.137 to 1.89 mA/cm^2^). The results indicate that the thermal oxide layer prevents electrons from moving into n^++^-poly-Si, and carrier collection was improved by removing the carrier-blocking thermal oxide layer. Furthermore, IQE was measured and the edge of the spectrum was about 1000 nm, indicating that the obtained *J*_SC_ was derived from the Si-QDs.

## Data Availability

All data supporting the conclusions of this article are included within the article.

## Abbreviations

EQE: External quantum efficiency
HRTEM: High-resolution transmission electron microscope
IQE: Internal quantum efficiency
*J*_SC_: Short-circuit current density
*J-V*: Current density–voltage
PECVD: Plasma-enhanced chemical vapor deposition
SIMS: Secondary ion mass spectroscopy
Si-QD: Silicon quantum dot
Si-QDML: Silicon quantum dot multilayer structure
TiO_*x*_:Nb: Niobium-doped titanium oxide
TOF-SIMS: Time-of-fright secondary ion mass spectroscopy
*V*_OC_: Open-circuit voltage
